# Transport and Use of Bicarbonate in Plants: Current Knowledge and Challenges Ahead

**DOI:** 10.3390/ijms19051352

**Published:** 2018-05-03

**Authors:** Charlotte Poschenrieder, José Antonio Fernández, Lourdes Rubio, Laura Pérez, Joana Terés, Juan Barceló

**Affiliations:** 1 Plant Physiology Lab., Bioscience Faculty, Universidad Autónoma de Barcelona, 08193 Barcelona, Spain; laura.perez.martin@uab.cat (L.P.); joana.tege@gmail.com (J.T.); juan.barcelo@uab.es (J.B.); 2 Department Biologia. Vegetal, Campus Teatinos, Universidad de Málaga, 29071 Málaga, Spain; ja_fernandez@uma.es (J.A.F.); lrubio@uma.es (L.R.)

**Keywords:** bicarbonate, transporter, metabolism, carbonic anhydrase, carboxylases, carbon concentration mechanisms, algae, seagrass, higher land plants, limestone soil

## Abstract

Bicarbonate plays a fundamental role in the cell pH status in all organisms. In autotrophs, HCO_3_^−^ may further contribute to carbon concentration mechanisms (CCM). This is especially relevant in the CO_2_-poor habitats of cyanobacteria, aquatic microalgae, and macrophytes. Photosynthesis of terrestrial plants can also benefit from CCM as evidenced by the evolution of C_4_ and Crassulacean Acid Metabolism (CAM). The presence of HCO_3_^−^ in all organisms leads to more questions regarding the mechanisms of uptake and membrane transport in these different biological systems. This review aims to provide an overview of the transport and metabolic processes related to HCO_3_^−^ in microalgae, macroalgae, seagrasses, and terrestrial plants. HCO_3_^−^ transport in cyanobacteria and human cells is much better documented and is included for comparison. We further comment on the metabolic roles of HCO_3_^−^ in plants by focusing on the diversity and functions of carbonic anhydrases and PEP carboxylases as well as on the signaling role of CO_2_/HCO_3_^−^ in stomatal guard cells. Plant responses to excess soil HCO_3_^−^ is briefly addressed. In conclusion, there are still considerable gaps in our knowledge of HCO_3_^−^ uptake and transport in plants that hamper the development of breeding strategies for both more efficient CCM and better HCO_3_^−^ tolerance in crop plants.

## 1. Introduction

Life on Earth is based on the photosynthetic transformation of inorganic carbon (C_inorg_) and water into energy-rich organic carbon (C_org_) compounds. In turn, these are oxidized by heterotrophs to obtain cellular energy, releasing again C_inorg_ in the form of CO_2_ into the atmosphere. Atmospheric CO_2_ is the main form of C_inorg_ assimilated by the terrestrial photosynthetic organisms. Dissolution of CO_2_ in water provides carbonic acid, which dissociates into bicarbonate (HCO_3_^−^) and carbonate (CO_3_^2−^). Ocean water contains about 90% of C_inorg_ in the form of HCO_3_^−^. It is calculated that, at preindustrial concentrations of atmospheric CO_2_, the seawater concentration of HCO_3_^−^ was 1757 µmol/kg. Even higher HCO_3_^−^ concentrations are currently observed due to increasing atmospheric CO_2_, which leads to acidification of the ocean and higher solubility of carbonate under these lower pH conditions. Photosynthetic marine organisms as well as submerged freshwater plants can use this abundant HCO_3_^−^ as a source for the biosynthesis of C_org_ [1,2,3]. 

Due to the weathering of limestone and dolomite, bicarbonate enters into the soil solution. The HCO_3_^−^ concentration in a solution phase should be controlled by the solubility of CaCO_3_. Calcite is the main carbonate mineral with an ion concentration product (Ksp) at 25 °C of 10^−8.35^ [4]. However, considerably higher HCO_3_^−^ levels than those predicted based on carbonate solubility constants may occur in soil solutions [5]. Biological activity contributes to HCO_3_^−^ build-up in soil solutions by hydrating CO_2_ from the atmosphere and from the respiratory activity of plant roots, microorganisms, and soil fauna. The CO_2_ hydration process catalyzed by soil carbonic anhydrase activity provided mostly by soil cyanobacteria and microalgae [6] can be considerably higher than the un-catalyzed process [7]. 

Terrestrial cyanobacteria can use the HCO_3_^−^ dissolved in the surrounding aqueous film for photosynthesis [8]. In contrast, high soil HCO_3_^−^ concentrations can injure the performance of higher land plants especially of the carbonate sensitive calcifuge species. In these calcareous soils with high pH, the availability of Fe and other essential micronutrients like Zn, Mn, and Cu is usually low due to precipitation as oxides or carbonates. This leads to the so-called lime-induced chlorosis and low yield in sensitive varieties of crops. Dicots such as citrus, deciduous fruit trees, vineyard, and legumes are the most sensitive mainly because of the interference of bicarbonate with their Fe acquisition mechanisms (strategy I). Grasses are less affected. Their Fe acquisition is based on phytosiderophore production (strategy II) [9]. Nonetheless, monocots like rice, maize barley, or wheat can be affected by severe Zn deficiency on carbonate-rich soils [10]. To what extent terrestrial higher plants are able to assimilate HCO_3_^−^—either soil-derived or of respiratory origin produced by soil microorganism and plant roots—is still under debate [11] and will be discussed below.

Heterotrophs, which are animals and humans, are net producers of CO_2_ by respiration. Bicarbonate is the main transport form of C_inorg_ from cells to the lungs where it is exhaled in the form of CO_2_. The carbonic acid/bicarbonate buffer is considered the most important system for cell pH homeostasis. Kidneys filter and reabsorb HCO_3_^−^. These processes are essential for the acid-base balance of the body [12]. Furthermore, HCO_3_^−^ transport plays an essential role in pH regulation during amelogenesis, which is the formation of enamel during tooth development [13], and in other biological calcification processes such as the development of reef cnidarians [14].

The presence of HCO_3_^−^ in all organisms opens the question of how this molecule is taken up, membrane-transported, and compartmentalized in these different biological systems. This review aims to give a comprehensive overview of the transport and metabolic processes related to HCO_3_^−^ in plants. Bicarbonate transport in cyanobacteria and human cells is much better documented and will be briefly presented for comparison.

## 2. Bicarbonate Transport

As an anion, HCO_3_^−^ is not freely permeable to the lipid bilayer of biological membranes. The presence of HCO_3_^−^ inside cells is either due to HCO_3_^−^ transport mediated by membrane transporter proteins or transmembrane diffusion of CO_2_ followed by fast transformation into HCO_3_^−^ using carbonic anhydrase (CA).

CO_2_ crosses biological membranes by diffusion either through the lipid bilayer or through pores. A subset of aquaporins and related proteins [15,16] can behave as CO_2_ channels [17]. Models based on artificial lipid bilayers indicated that the resistance for CO_2_ diffusion is small and mostly limited by unstirred layers. According to the authors, an estimated permeability of 3.6 cm s^−1^ makes it unlikely that CO_2_ is transported through aquaporins or other transporter proteins [18]. Contrastingly, studies on real bio-membranes provided clear evidence that membranes can offer resistance to CO_2_ diffusion and that this resistance depends on the membrane’s protein composition [19]. There is now functional evidence that some, but not all, plant aquaporins can enhance CO_2_ diffusion into both stomatal guard cells and mesophyll cells [20,21]. However, the relative contribution of both CO_2_ diffusion pathways to the downhill, non-energized movement of CO_2_ through the membranes of cyanobacteria, eukaryotic algae, and embryophytes has to be further clarified [22]. Transport of HCO_3_^−^ through aquaporins has not been shown. As a charged chemical species, the HCO_3_^−^ ion is submitted to electrochemical gradients that govern plasma membrane ion transport. Since cyanobacteria, algae, and plant cells have an inside negative membrane potential (E_m_), the uphill HCO_3_^−^ uptake must be energized. It takes place through transporters in cyanobacteria [23], microalgae [24,25], macro algae [26], and seagrasses [27]. The plasma membrane downhill HCO_3_^−^ efflux takes place through anion channels. The only available evidence for an HCO_3_^−^ permeable anion channel is the R-type, which has been found in the hypocotyls of *Arabidopsis thaliana* with a selectivity sequence for anions. NO_3_^−^ (2.6) > SO_4_^2–^ (2.0) > Cl^−^ (1.0) > HCO_3_^−^ (0.8) >> malate^2−^ (0.03) [28,29]. In addition, the inner chloroplast envelope protein LCIA of *Chlamydomonas reinhardtii* has been proposed to be an HCO_3_^−^ permeable channel [30,31,32]. Furthermore, Raven et al. [33] have proposed the existence of HCO_3_^−^ permeable anion channels in the thylakoid membrane as an element of the carbon concentration mechanisms (CCM) in microalgae.

### 2.1. Bicarbonate Transport by Solute Carriers (SLC) in Humans and Mammals

Most bicarbonate transporters belong to the **s**olu**t**e **c**arriers (SLC), which is a large group of secondary active membrane transporters for relatively small molecules. The best studied bicarbonate transporters are SLC in humans due to severe diseases related to the malfunctioning of these transporters [34,35]. In humans, 430 members organized in 52 families have been identified [36]. Proteins transporting HCO_3_^−^ belong to the families SLC26 (Sulfate permease SulP) and SLC4. The phylogenetically ancient gene family SLC26 encodes for multiple anion exchangers and channels. Some are relatively ion specific, but others have a broad substrate range. Besides transporting inorganic anions like HCO_3_^−^, Cl^−^, SO_4_^2−^, and I^−^, oxalate and formate may be transported. Structural models indicate that these polypeptides have 10 to 14 membrane-spanning domains flanked by a cytoplasmic *N*-terminal and a cytoplasmic C-terminal bound to a STAS (sulphate transporter anti sigma factor-like) domain [37]. The gene family *SLC4* contains genes that code for proteins transporting HCO_3_^−^ or the closely related CO_3_^2−^ along with a monovalent anion (Cl^−^) or cation (Na^+^) [38]. These proteins have 14 transmembrane spanning domains grouped into a 7 + 7 inverted repeat topology.

**Figure 1:**
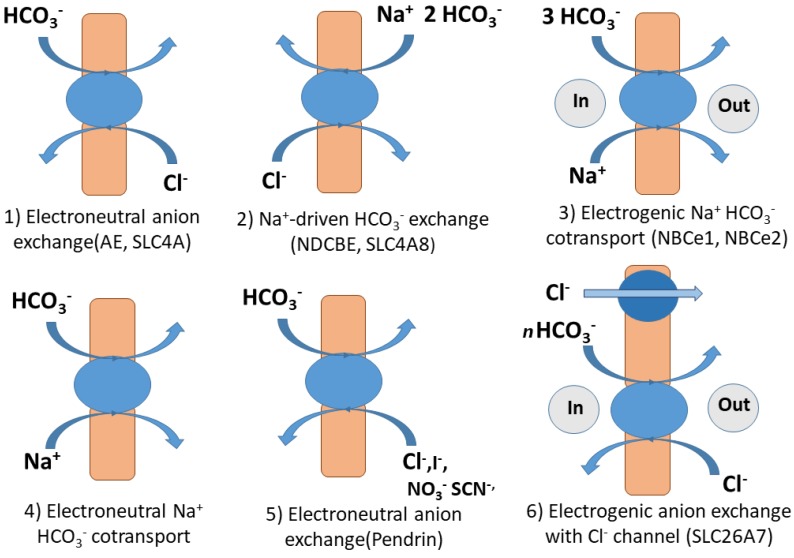
Mechanisms of HCO_3_^−^ transport by solute carriers (SLC) in humans and mammals drawn with information from [37,40].

Different ways for HCO_3_^−^ transport through the membranes in mammalian and human cells can be distinguished (see Figure 1). The first includes electroneutral, Na^+^- independent anion exchange between HCO_3_^−^ and Cl^−^ using anion exchange transporters (AE) encoded by genes of the SLC4A family. The second includes sodium-driven Cl^−^/HCO_3_^−^ exchanger (NDCBE) encoded by SLC4A8. This transporter is thought to exchange 1 Cl^−^ for 2 HCO_3_^−^ and 1 Na^+^. The next transport mechanism comprises electrogenic Na^+^/HCO_3_^−^-cotransport performed by NCBT transporter proteins NBCe1 and NBCe2 encoded by SLC4A4 and SLC4A5. The fourth way is the electroneutral Na^+^- HCO_3_^−^ cotransport or Na^+^-driven Cl^−^/HCO_3_^−^ exchange through the transporter protein encoded by SLC4A10 [39,40,41]. The fifth mechanisms involves electroneutral Cl^−^/HCO_3_^−^ exchangers that also can exchange either I^−^, NO_3_^−^, SCN^−^, or formate encoded by SLC26A (Pendrin) or NO_3_^−^, OH^−^, SO_4_^2−^, oxalate, and formate (SLC26A6). (Electrogenic Cl^−^/HCO_3_^−^ exchange, with channel activity for Cl^−^, SO_4_^2−^, and oxalate (SLC26A7) and Electrogenic Cl^−^/HCO_3_^−^ exchange with Cl- channel activity, NaCl cotransport or Cl^−^ -independent HCO_3_^−^ transport (SLC26A9) are also HCO_3_^−^ transport mechanisms [35] (see Figure 1). 

### 2.2. C_inorg_ Transporters in Cyanobacteria 

Five modes of C_inorg_ transport have been described in cyanobacteria. (i) BCT1 is an inducible high affinity (K_0.5_ for HCO_3_^−^ ≈ 15 µM) transporter located in the plasma membrane that belongs to the ATP binding cassette (ABC) transporter family [42] although transport energization by ATP consumption has not been proven [43]. BCT1 is a multi-meric complex composed by four subunits. CmpA is located in the periplasmic space and binds HCO_3_^−^ with a very low K_0.5_ of 5 µM [44] and also binds Ca^2+^ as a cofactor [45]. CmpB is a dimer within the plasma membrane and CmpC and CmpD are extrinsic proteins that share binding sites for ATP. CmpC has an extra domain involved in the allosteric regulation of BCT1 similar to the NrtC protein of NRT1 transporter. In this later case, the domain of the NrtC protein is involved in the inhibition of transport in the presence of NH_4_^+^ [46]. BCT1 is found in ß-cyanobacteria but absent in marine cyanobacteria. However, it is present in the α-cyanobacteria Synechococcus WH5701, which can live in a wide range of C_inorg_ concentrations and salinities [43].

(ii) SbtA is a low C_inorg_-inducible, high affinity (K_0.5_ 2–5 µM), plasma membrane HCO_3_^−^ transporter that uses Na^+^ as a driving ion with a half saturation constant around 1 mM for this ion [47]. Although initially considered a single unit-type transporter, it has a bigger complex size, which suggests that, in its functional form in the plasma membrane, this transporter is a tetramer [48]. It has been suggested that SbtA is activated by a serine-threonine protein kinase [49] that also depends on Na^+^ [50]. SbtA homologs seem to be present in many ß-cyanobacteria although this has only been confirmed in Synechocystis PCC6803 [47] and Synechococcus PCC7002 [50].

(iii) BicA HCO_3_^−^ transporters are also dependent on Na^+^. Their affinity for HCO_3_^−^ transport with a K_m_ ranging from 74 µM to 353 µM (1.7 mM for Na^+^) is lower than that of SbtA. Nonetheless, BicA is able to maintain a high flow of C_inorg_ for photosynthesis. BicA transporters are expressed at low levels under conditions of high CO_2_ but they are highly inducible under low CO_2_. They have been discovered in the coastal marine cyanobacterium Synechococcus PCC7002 [51] and they are present in both α-cyanobacteria and ß-cyanobacteria. BicA transporters belong to the large family of prokaryotic and eukaryotic transporters often described as sulphate, SulP family transporters. The C-terminus includes a hydrophilic STAS domain (see also Section 2.1) involved in the allosteric regulation that has also been found in *A. thaliana* sulfate transporters [52].

(iv) NDH-I4 is a constitutive protein complex located in the plasma membrane that accelerates CO_2_ uptake. The passive entry of CO_2_ is followed by the conversion (NDH-I mediated) to HCO_3_^−^ [53,54].

(v) NDH-I3 is a second, complex, low CO_2_ -inducible system involved in CO_2_ uptake located in the thylakoid membrane. It works in a similar manner to NDH-I4 [53,54].

### 2.3. C_inorg_ Transport in Microalgae

*C. reindhardtii* uptake of C_inorg_ has been associated with the activity of an ATP-binding cassette transporter, HLA3, and the homolog of a formate-nitrite transporter LCIA that is also called NAR1.2 [30]. HLA3 is located in the plasma membrane and LCIA in the chloroplast envelope. The absence of LCIA decreases the amount of HLA3 mRNA, which indicates a regulation by the chloroplast-encoded LCIA of the expression of HL3 encoded in the nuclear genome [31]. While the HCO_3_^−^ transport mechanism of HL3 seems to be clear, LCIA has been proposed to be an HCO_3_^−^ channel [22,30,31]. If so, HCO_3_^−^ ions would be transported through such a channel downhill and could not accumulate HCO_3_^−^ over the equilibrium prediction. However, the addition of mM concentrations of HCO_3_^−^ to *Xenopus laevis* oocytes expressing *NAR1.2* evokes a membrane depolarization as does the addition of mM concentrations of NO_2_^−^, which suggests an HCO_3_^−^ transport into the chloroplast by H^+^ symport instead of the transport through a channel. This mechanism would also be consistent with the need to overcome the electrochemical gradient for HCO_3_^−^ in the stroma relative to the cytosol [55]. The ycf10 is also related to C_inorg_ transport. Disruption of the plastid *ycf10* inhibits the C_inorg_ accumulation in the chloroplast. Its gene product known as the protein CemA was originally proposed as a C_inorg_ transporter, but its similarities with the cyanobacterial PxcA involved in Na^+^-dependent H^+^ extrusion suggest that CemA may play a similar role in the energization of the chloroplast envelope [55]. The HCO_3_^−^ uphill transport through the plasma membrane and the chloroplast envelope agrees with the early observation of a vanadate sensitive C_inorg_ transport at both levels. A second C_inorg_ transporter proposed for the plasma membrane in *C. reindhardtii* is LCI1 [56,57]. The overexpression of this protein increases the affinity for C_inorg_ and enhances HCO_3_^−^ uptake. The protein is encoded by an orphan gene [55] and does not have any known functional motif. The proteins CCP1/2 have also been proposed to take part in C_inorg_ uptake by the chloroplasts. They show similarities with the mitochondrial carrier proteins superfamily, but knock-outs of CCP1/2 do not show defects in photosynthesis [58]. Thus the specific role of CCP1/2 proteins in C_inorg_ transport has yet to be clarified.

The active uptake of HCO_3_^−^ was described for natural populations of marine phytoplankton dominated by large diatoms [59]. However, the HCO_3_^−^ transport mechanisms at the molecular level have been studied in the model diatom species *Phaeodactylum tricornutum* and *Thalassiosira pseudonana* [60,61]. In *P. tricornutum*, ten putative HCO_3_^−^ transporters have been identified. They are similar to the unrelated SLC4 and SLC26 mammalian protein families (see Section 2.1). SLC4 has been characterized as a HCO_3_^−^ transporter in the plasma membrane of *P. tricornutum* and SLC4 homologs have also been found in *T. pseudonana* [25]. Photosynthesis in the diatom species is sensitive to 4,4′-diisothiocyanatostilbene-2, 2′-disulfonic acid (DIDS), which is an inhibitor of anion exchange, and depends on the presence of Na^+^ in the medium (K_0.5_ 28 mM, saturation at 100 mM Na^+^). This suggests the existence of an HCO_3_^−^ uptake mechanism based on Na^+^ symport or on a Na^+^ dependent Cl^−^/HCO_3_^−^ anti-port [25]. A different group of SLC4 transporters located in the chloroplast envelope have been proposed for transporting HCO_3_^−^ to the chloroplast stroma [25,60,61]. The active transport rate of dissolved inorganic carbon through the chloroplast envelope is ten-fold that of HCO_3_^−^ transport across the plasmalemma [54]. However, further investigations are required to elucidate the molecular identity of the protein and the transport mechanism in the context of the complex four-layer chloroplast envelope of diatoms [62].

In micro-algal species, genetic tools are still not available and HCO_3_^−^ uptake has been revealed by physiological methods that include the photosynthetic sensitivity to inhibitors of external CA, pH buffers to dissipate electrochemical H^+^ gradients, and inhibitors of anion exchangers. Therefore, a direct entry of HCO_3_^−^ has been proposed for the marine eustigmatophycean *Nannochloropsis gaditana* [63,64,65]. The absence of external CA and the sensitivity to DIDS suggest an anion exchange mechanism for HCO_3_^−^ transport. A DIDS and 4-acetamido-4′-isothiocyanato-stilbene-2, 2′-disulfonic acid (SITS) sensitive photosynthesis has been described in *Eminliania huxleyi* [66]. SITS is the putative inhibitor of the anion exchanger 1 (AE1), which works as a Cl^−^/HCO_3_^−^ antiporter in red blood cells [67] (see Figure 1). In contrast, a DIDS/SITS insensitive HCO_3_^−^ transport has been described for *Dunaliella tertiolecta* [68].

### 2.4. C_inorg_ Transport in Macroalgae 

One of the first examples for the use of HCO_3_^−^ in macro-algae was described in the giant inter-nodal cells of Characeae living in alkaline media [69]. The active efflux of H^+^ through the putative H^+^-ATPase causes a local acidification of the apoplast in about two pH units [69]. The presence of CA activity in the acidic zones accelerates the conversion of HCO_3_^−^ to CO_2_ that diffuses across the plasmalemma [70]. The cytosolic pH homeostasis requires the presence of alkaline areas between the acid zones, which produces the spectacular banding observed in these organisms under the light [71]. An alternative mechanism for HCO_3_^−^ use and hence for banding was given by Lucas et al. [72]. These authors, by using quasi apoplastic pH measurements in flow- through experiments, provide evidence for an H^+^/HCO_3_^−^ symport in the acid bands in which the electrochemical proton gradient generated by the H^+^-ATPases is secondarily used for HCO_3_^−^ transport. According to the model by Walker et al. [69], the alkaline zones are needed for compensating cytosolic pH through OH^−^ efflux, which originated via the catalyzed cytosolic dehydration of HCO_3_^−^. A similar model to the one proposed by Walker et al. [69] has been described for freshwater flowering plants where the acid zone is the abaxial (lower) leaf surface and the alkaline zone is the adaxial (upper) leaf surface [22,73].

The use of HCO_3_^−^ as a source of inorganic carbon for photosynthesis has been described for the majority of marine macro-algae and seagrasses [74,75,76]. The most common mechanism of HCO_3_^−^ use is the apoplastic conversion to CO_2_, which is shown in *Condrus chrispus* [77], *Porphyra leucosticta* [78], a series of red macroalgae [79], and *Phyllariopsis puspurascens* [80]. More information is available in References [33,75,81,82]. Alternatively, other algal species have been described as direct HCO_3_^−^ users. Most of the evidence for a direct uptake of HCO_3_^−^ ions comes from experiments in which the inhibitors of anion exchanger, mainly DIDS and SITS, are used to inhibit HCO_3_^−^ transport and, therefore, photosynthesis [82]. Larsson and Axelson [83] examined 11 green, 5 red, and 11 brown macro algae. Photosynthesis was DIDS-sensitive only in Chaetomorpha, Monostroma, and ulvaceans (Ulva and Enteromorpha), but not in the rest of green, red, or brown algae tested. More information is available in Reference [84]. Fernández et al. [26] show a DIDS-sensitive anion exchanger as the main mechanism for HCO_3_^−^ uptake in the giant kelp *Macrocystis pyrifera*. DIDS-sensitivity has also been reported in the red algae *Eucheuma denticulatum* [85] while a residual DIDS-sensitive photosynthetic activity was found in *Gracilaria gaditana* [86]. Calculations made from photosynthetic conductance were used to suggest direct HCO_3_^−^ uptake in *Laurencia pinnatifida* [87].

### 2.5. C_inorg_ Transport in Seagrasses

Seagrasses have been described as HCO_3_^−^ users [82,88,89,90,91]. Based on the lack of photosynthesis inhibition in seagrasses by DIDS and SITS, Larkum et al. [91] hold that HCO_3_^−^ influx through anion exchangers does not take place in the leaves of seagrasses. These substances inhibit AE1 that are present in algae but in angiosperms (including marine) DIDS and SITS have been described as inhibitors of anion channels [29,92] that may have a distinct role in plasma membrane anion transport [87]. As an alternative, Larkum et al. [91] suggest a proton symport as the mechanism for direct uptake of HCO_3_^−^. Such a mechanism has been proposed for *Zostera marina* [88,93,94], *Zostera noltii* [95], *Posidonia oceanica* and *Cymodocea nodosa* [96], *Halophila stipulacea* and *Ruppia maritima* [96], *Ruppia cirrhosa* [97], and *Halophila ovalis* [98]. Based on the response of seagrasses to acetazolamide (AZ) and TRIS buffers, Beer et al. [88] suggest three mechanisms for carbon acquisition. First, an apoplastic dehydration of HCO_3_^−^ catalyzed by CA and the subsequent diffusion of CO_2_ across the plasmalemma. This mechanism is proposed for plants that show AZ-sensitive TRIS-insensitive photosynthesis, but the ubiquitous presence of plasmalemma H^+^-ATPase cannot be ignored [99]. Second, the catalyzed apoplastic dehydration of HCO_3_^−^ to CO_2_ in acid regions generated by the activity of the H^+^-ATPases. This mechanism would be sensitive to AZ and TRIS. Third, the direct uptake of HCO_3_^−^ ions by symport with H^+^. In this case, the electrochemical gradient for H^+^ generated by the activity of the plasmalemma H^+^-ATPases drives the direct HCO_3_^−^ transport. This mechanism would be AZ-insensitive and TRIS-sensitive. The two first mechanisms involve apoplastic accumulation of OH^−^ and CO_2_ diffusion across the plasmalemma and the third one implies accumulation of HCO_3_^−^ and likely OH^−^ in the cytosol (see Table 1). In contrast to humans (see Figure 1) and cyanobacteria (Section 2.2), no Na^+^ -dependent HCO_3_^−^ uptake system has been reported in plants. The only example for a Na^+^-driven ion transport system is the high affinity transporter for NO_3_^−^ and P_i_ in the seagrass *Zostera marina* [100]. In that case the electrochemical gradient for Na^+^ is maintained because of very low membrane permeability for Na^+^ and the action of a Na^+^/H^+^ antiporter, which is similar to the SOS1 present in terrestrial vascular plants [101].

The availability of the genome of *Zostera marina* [103] allows the *in silico* search for genes potentially involved in C_inorg_ transport. Using the web application Phytozome (http://www.phytozome.net), which is a comparative platform for green plant genomics [104], we searched for genes with homologies with the HLA3 transporter of *C. reinhardtii* and SLC4 transporter of *P. tricornutum*. The search for homologies in the genome of *Z. marina* with *ChHLA3* sequence results in six genes with high homology, all of them listed as ABC transporters. In contrast, the search for homologies with *PhSLC4* results in five sequences of medium-to-low homologies with genes encoding boron transporters and anion exchangers. The public availability of the genome of seagrasses will be a valuable tool for the future investigation of the exact molecular identities of C_inorg_ transporters, cellular location, mechanism, kinetic properties, and regulation.

### 2.6. C_inorg_ Transport in Higher Land Plants

While HCO_3_^−^ transporters are already quite well-characterized in cyanobacteria, algae, and mammals, the information on higher vascular land plants is scarce. Seven loci of genes coding for transporters of the HCO_3_^−^ family are listed in the gene databases of the genetically well-characterized *A. thaliana*. The best studied protein is BOR1. This protein belongs to the solute carrier family type SLC4 and presents homology to SLC4A1, which is the band 3 transporter highly abundant in erythrocytes. As SLC4A1, BOR1 has a gate and a core domain and acts with an elevator mechanism. However, BOR1 has an inward rotated core domain providing an occluded state, which suggests that it may undergo structural transitions allowing access from either side of the membrane [105]. Bicarbonate transport by Band 3 is a unidirectional pathway out of the erythrocyte. A further substantial difference is that BOR1 is an efflux-type borate transporter responsible for root-to-shoot transport of this essential plant nutrient. BOR1 is located in the xylem parenchyma cells and loads borate into the xylem, which is then transported to shoots by the transpiration stream [106]. The other six genes code for BOR2 to BOR7 [107]. All seem to be involved in the transport of borate or boric acid rather than in HCO_3_^−^ transport.

Although no selective HCO_3_^−^ transporters or channels have so far been characterized in higher land plants, the possibility of membrane transport by specific or unspecific anion transporting proteins cannot be excluded. Several studies provide indirect support for HCO_3_^−^ uptake by plant roots. Under exposure to high HCO_3_^−^ (5 mM to 20 mM), a strong inhibition of nitrate, sulphate, and phosphate uptake by roots has been observed [108]. Such inhibition could be caused, at least in part, by competition between HCO_3_^−^ and other anions for transport mechanisms with low anion specificity. An electrophysiological approach to ion selectivity of a voltage-dependent anion channel in *A. thaliana* hypocotyls revealed low but reproducible HCO_3_^−^ currents. A permeability ranking of NO_3_^−^ ≥ SO_2_^4−^ > Cl^−^ > HCO_3_^−^ >> mal^2−^ could be established [28]. More recently, such a channel with permeability for several anions has been identified as QUAC1/ALMT12, which is a channel that releases anions from guard cells [109]. In fact, anion channel currents in plants have mainly been studied in guard cells where they contribute to the mechanisms for controlling stomatal resistance (see Section 3.3). Slow Anion Channels (SLACs) and Quick Anion Channels (QUAC) are involved in the transport of NO_3_^−^ and Cl^−^ (SLAC1), NO_3_^−^ (SLAH3), or malate (QUAC1/ALMT1) [110]. QUAC1/ALMT12 is activated by xylem derived extracellular SO_4_^2−^ [111].

Anion channels in roots are less characterized. In *A. thaliana* roots, a *slah3-1* mediated Cl^−^ and possibly NO_3_^−^ efflux in response to ABA has been shown [112]. Recently, Canales et al. [113] reported comparative root expression profiles at a cell resolution level for anion channels in *A. thaliana*. Nitrate channel SLAH3 was strongly expressed in the mature root zone while the Voltage Dependent Anion Channel (VDAC1) was localized to the meristem zone. VDACs 2 and 4 have been reported expressed in all plant organs [114]. At the subcellular level, VDACs are localized at the outer mitochondrial membrane and in small vesicles located in the cell periphery [115]. The ion selectivity of VDACs depends on ionic strength. Higher selectivity for Cl^−^ is achieved with lower ionic strength [116].

It has been stated that, on limestone soils, HCO_3_^−^ can passively enter into plant roots. Then it is long–distant transported via xylem vessels to the leaves where, after transformation by CA anhydrase, the resulting CO_2_ can be assimilated along with the atmospheric CO_2_ [11]. The apoplastic, passive radial transport pathway in the roots is disrupted at the endodermal level due to the hydrophobic Casparian strip. Therefore, to reach the vascular cylinder, a substance has to first pass through the plasma membrane into the symplasm. This implies either a still unidentified HCO_3_^−^ membrane transport system or the conversion of HCO_3_^−^ into CO_2_, which may easily diffuse into the stele. Apoplastic by flow, either through the young root tips where the Casparian strip has still not fully developed or at sites where lateral root emergence from the pericycle disrupts this hydrophobic barrier, may be another way HCO_3_^−^ enters the stele. Contribution of this apoplastic bypass is relatively small in the case of NaCl [117] or Cd [118]. We could not find specific data for HCO_3_^−^.

Early investigations using ^11^C or ^14^C isotopes as markers for HCO_3_^−^ provided evidence for uptake of HCO_3_^−^ by roots and transport to the shoots [119,120,121,122]. However, the contribution of C_inorg_ taken up by roots may be less than 1% taken up by leaves [123]. The ^14^C from labelled H^14^CO_3_^−^ supplied through the roots was found to be incorporated into sugar, starch, and proteins of leaves [124]. As plants can acquire C_inorg_ from different sources including atmospheric CO_2_ and respiratory CO_2_, the experimental design is critical. Solution pH used for supplying labelled HCO_3_^−^ to the plants deserves special attention. At pH 8, most of the labelled C_inorg_ is in the form of HCO_3_^−^ but a small percentage of labelled CO_2_ can be present and CO_2_ diffusion into the root cells may occur, which will be followed by transformation of this CO_2_ into labelled HCO_3_^−^ by CA. This transformation can be even more relevant considering that the pH of cell walls and xylem sap are usually in the acid range. The pH of the leaf apoplast of sunflowers remained stable around 6.4 to 6.5 even if roots were exposed to 10 mM HCO_3_^−^ [125]. However, it has to be taken into account that apoplast alkalinization is a general response to stress in plants [126]. Enhanced Cl^−^ supply under stress causes alkalization of the root apoplast due to the symport of 2 H^+^ per 1 Cl^−^ [127]. Increasing the external pH of the root bathing solution also increases pH of both *A. thaliana* root cell walls [128] and xylem sap [129]. This favors HCO_3_^−^ over CO_2_ formation. Nonetheless, even under severe stress conditions, such as drought or fungal infection with a strong alkalinization effect in the apoplast, the increased pH values remain nearly neutral [130]. Therefore, in plants with their roots exposed to HCO_3_^−^, the proportion of HCO_3_^−^ over CO_2_ during radial transport of C_inorg_ from soil to the stele and within the xylem sap up to the leaves may be considerably lower than in the soil solution surrounding the plant roots. The direct use of root-derived HCO_3_^−^ by CA in chloroplasts to supply CO_2_ for Rubisco is unlikely when taking into account the low chloroplast permeability of HCO_3_^−^ (1 × 10^−8^ m s^−1^) in comparison to CO_2_ (range from 2.3 × 10^−4^ to 8 × 10^−4^ m s^−1^), which was recently shown by mass inlet mass spectrometry (MIMS) using ^18^O labelled C_inorg_ [131]. 

## 3. Formation and Use of Bicarbonate in Plants

As seen in higher plants, no selective HCO_3_^−^ transporter or channel has been characterized at the molecular level. Membrane transport of HCO_3_^−^ in these organisms is still unclear. Contrastingly, the contribution of HCO_3_^−^ to essential metabolic pathways and the total assimilation of C_inorg_ in plants is reliably documented. 

**Figure 2:**
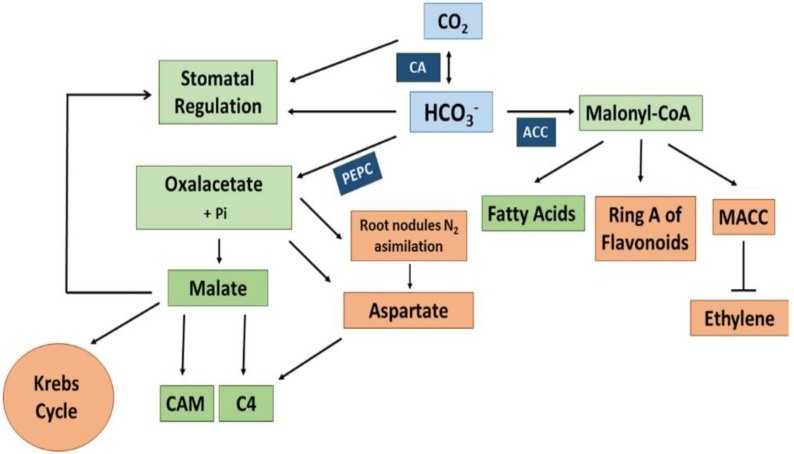
Metabolic pathways related to three major plant carboxylases using HCO_3_^−^ as substrate. CA, carbonic anhydrase; PEPC, phosphoenolpyruvate carboxylase; ACC, acetyl-CoA carboxylase; MCC, malonyl-1-aminocyclopropane-1-carboxylic acid.

The CA-generated HCO_3_^−^ serves as a substrate for different carboxylases among others acetyl-CoA carboxylase (ACCase, EC.6.4.1.2) and phosphoenolpyruvate carboxylase (PEPC, EC 4.1.1.31). ACCase contains a biotin carboxylase, a biotin carboxyl carrier protein, and a carboxyl transferase. It catalyzes the carboxylation of acetyl-CoA to malonyl-CoA in the chloroplast and the cytosol [132]. Malonyl-CoA is the precursor for fatty acid formation and elongation. Moreover, it participates in the biosynthesis of ring A of flavonoids through the polycetic pathway and in the biosynthesis of malonylated aminocyclopropane-1-carboxylic acid (MACC), which is involved in the down-regulation of ethylene production in plants (see Figure 2). Other biotin-containing carboxylases operating with HCO_3_^−^ are 3-methylcrotonyl-CoA carboxylase, which is involved in the mitochondrial pathway of leucine catabolism. Geranyl-CoA carboxylase likely works in the metabolism of cyclic terpenes [133,134]. 

PEPC plays a major role in the carbon assimilation processes in plants. This enzyme in the presence of Mg^2+^ or Mn^2+^ ions catalyzes the β-carboxylation of phosphoenolpyruvate (PEP) yielding oxalacetate (OAA) and inorganic phosphate (P_i_) in an irreversible reaction (see Figure 2). 

The relative importance of the contribution of HCO_3_^−^ to the total plant C_org_ as well as the assimilation mechanisms and their consequences for plant adaptation to different environmental conditions depend on the plant species and the characteristics of the habitat (see Section 3.2 and Section 4). In all cases, the cooperation of the two enzymes, CA and, in higher plants, PEPC, is essential.

### 3.1. Plant Carbonic Anhydrase and Phosphoenolpyruvate Carboxylase 

Carbonic anhydrases (CAs, EC 4.2.1.1) are metallo-enzymes that catalyze the reversible hydration of CO_2_ forming HCO_3_^−^. Zinc is the required metal at the catalytic site for CA activity. Some exceptions are several coastal diatoms with cadmium–containing CA (CDCA). The Cd^2+^ at the catalytic site is fully exchangeable for Zn^2+^ [135]. The natural use of Cd^2+^ in this ζ-CA class enzyme is considered an evolutionary adaptation to low Zn^2+^ availability in marine habitats [136]. 

CA enzymes are ubiquitous in nature (animals, plants, archaebacteria, and eubacteria) and are an example of convergent evolution. Based on sequence comparison, CA proteins are grouped into seven distinct classes: α, β, γ, δ, ζ, η, and θ-CAs [137,138,139,140]. In higher land plants, only α, β, γ CAs are found. The δ and ζ classes are restricted to marine diatoms and η-CA so far has only been reported in *Plasmodium falsiparum* [141]. θ-CA seems more widely distributed in algae and cyanobacteria [142] and it has been reported critical for photosynthesis in the diatom *Phaedactylum tricornutum* [143]. The ubiquity of the distribution of CAs implies that they play diverse and essential roles in many biological processes. They have been related to respiration and transport of CO_2_/HCO_3_^−^ between tissues, pH and CO_2_ homeostasis, electrolyte secretion in a variety of tissues/organs, various biosynthetic reactions, and CO_2_ fixation [142,144]. In addition, CA is a plausible source of hydrogen sulphide (H_2_S) within plant leaves by catalyzing the conversion of carbonyl sulphide (COS) to CO_2_ and H_2_S [145].

Higher plants contain three evolutionarily distinct CA families including αCAs, βCAs, and γ CAs where each family is represented by multiple isoforms in all species [142,146,147]. Alternative splicing of CA transcripts is common. Consequently, the number of functional CA isoforms in a species may exceed the number of genes [147]. CAs are expressed in numerous plant tissues and in different cellular locations. The most prevalent CAs are those in the chloroplast, cytosol, and mitochondria. CAs have been found in the thylakoid lumen of Chlamydomonas and Phaeodactylum. They are an important component of the CCM in these species and, therefore, essential for photosynthesis and growth [143,148]. This diversity in location is paralleled in the many physiological and biochemical roles that CAs play in plants [142,147,149,150]. As in humans and animals, many of these roles are related to the CA-driven regulation of cell pH, which, in turn, can participate in multiple regulatory processes through electrical signals, changes in cytosolic Ca^2+^ concentrations, and plant hormones [150,151,152] among others.

#### 3.1.1. Plant α-Carbonic Anhydrases (αCA)

*Arabidopsis thaliana* contain eight αCA (AtαCA1-8) [153]. Genes for αCa1, αCA2, and αCA3 are expressed in green and reproductive tissue (stems, rosette leaves, caulinar leaves, and flowers). Only *αCA2* presents root expression. While expression of *αCA1* is independent of the level of CO_2_, the expressions of *αCA2* and *αCA3* are induced under conditions of low CO_2_ concentrations [149]. *αCa1* is expressed in chloroplasts and *αCA2* is expressed in the plasma membrane. α-CA4 is implicated in the processes leading to energy dissipation in the PSII antenna [154]. Arabidopsis *αCA8* is clearly a pseudogene since it encodes in-frame stop codons [147]. Tissue-specific expression has also been reported for other species. In sorghum, the αCA Sb5G039000 is expressed specifically in anthers while, in the legume species *Medicago truncatula* αCAs Mt1g059900 and Mt1g059940, are expressed in root nodules [147]. There is increasing evidence that αCAs can play an important role in photosynthesis [150]. Under conditions of increasing light intensity, the expression of *αCA2* decreases while the expression of *αCA4* increases. Knock-out mutants of these chloroplast-located αCAs exhibit contrasting responses in comparison of the wild type. Both the quantum yield at photosystem 2 (PS2) and the electron transfer to O_2_ decreased while non-photochemical quenching (NPQ) and CO_2_ assimilation were enhanced in plants lacking αCA2. The opposite was observed in *αCA4* knock-outs [155]. The authors hypothesize that these αCAs may participate in the regulation of H^+^ flux into the PS2 protein PsbS, which regulates qE-type NPQ. 

#### 3.1.2. Plant β-Carbonic Anhydrases

βCAs are most abundant in land plants where they participate in photosynthesis [147]. *Arabidopsis thaliana* has six βCAs [147]. *βCAs* genes are highly expressed in leaf tissue. Expressed sequence tag experiments revealed that *βCA1* to *βCA6* are expressed in rosette leaves, caulinar leaves, and flowers. *βCA3* is also strongly expressed in reproductive tissue while *βCA4*, *βCA5*, and *βCA6* are expressed in all tissues including roots. βCAs have been found in chloroplasts, mitochondria, the cytosol, and the plasma membrane [144,149]. Targeting analysis using green fluorescent protein fusion proteins confirmed the subcellular localization of plant βCAs: *βCA1* and *βCA5* are expressed in chloroplasts while βCA2 and βCA3 are cytosolic. Isoforms βCA4, βCA4.1, are localized in the plasma membrane while the short form, βCA4.2, is cytosolic. βCA5 and βCA6 are localized in the chloroplast and mitochondria, respectively [149]. 

The role of βCAs in photosynthesis of land plants seems especially relevant in grasses with C_4_-type photosynthesis [156] or for plants under limited C_inorg_ supply (see Section 3.2). Carbonic anhydrases could be versatile. They may be involved not only in photosynthesis and responses to CO_2_ and light but also in seed germination, morphogenesis, nodule development, and responses to abiotic stress [157,158]. The tobacco salicylic acid-binding protein 3 (SABP3) is a chloroplast βCA that exhibits antioxidant activity and plays a role in the hypersensitive defense response [159]. Furthermore, *β*CA1 is related to ethylene signaling responses, photosynthetic performance of cotyledons, and Arabidopsis seedling survival [160]. 

#### 3.1.3. Plant γ-Carbonic Anhydrases

Plant γCAs are codified in the nucleus but localized in mitochondria [139]. So far, no higher plant γCA with CA activity has been identified. Nonetheless, plant proteins with the active-site residues found in γCAs from archaebacteria and cyanobacteria have been found. In *A. thaliana*, five γCA- related genes have been reported including three *γCA* genes and two genes encoding γCA-like proteins. In contrast to γCA proteins, the γCA-like proteins do not have the required Zn-coordinating amino acid residues. Plant *γCA* genes encode for a part of the mitochondrial Complex I (NADH-ubiquinone oxidoreductase). Complex I knock-out lines present adverse effects: non-viable seeds, high levels of mitochondrial Complexes II and IV, and the alternative oxidase. However, this is in contrast with reduced levels of photosynthetic proteins [161]. A proteomic approach has recently found enhanced γCA root levels during the induction phase of Al-tolerance in the hyper-resistant grass *Urochloa decumbens*. This increase occurred along with higher adenylate kinase activity and supports a role for γCA in the maintenance of ATP-production during the Al tolerance response [162].

#### 3.1.4. Plant PEP Carboxylases

Phosphoenolpyruvate carboxylases (PEPC) are located in the cytosol and catalyze the β-carboxylation of PEP to oxaloacetate using HCO_3_^−^ in an irreversible process. The OAA can then be reduced through NADH or NADPH-dependent malate dehydrogenase to malate in a reversible process. PEPCs are present in bacteria, algae, and plants. The typical plant PEPC (class 1 PEPC) has four identical subunits of 107 kDa. Multiple isoforms have been identified in leaves of C_3_, C_4_, and CAM plants [163,164,165]. In *Sorghum bicolor*, which is a plant with C_4_-type photosynthesis, five PEPC genes (*PEPC1-5*) have been identified. The plant PEPC is highly regulated. Phosphorylation through PEP carboxykinase (PEPCK) at the *N*-terminal phosphorylation domain [166] and allosteric regulation by glycine and glucose-6-P enhances the activity. Inhibition is achieved by both allosteric regulation especially by malate and by ubiquitination [167]. In addition to the typical plant-type PEPC, plants also contain a bacterial-type PEPC (BPEPC) of 118 kDa [153]. The BPEPC is highly expressed in floral tissues as well as in seeds and fruits. It has recently been shown that high BPEPC occurs in tissues that accumulate high malate concentrations [168]. There is a tight interaction between PTPC and BTPC, which forms the class 2 PEP. This is an enzyme complex that, in contrast to class 1 PEPC, is mostly insensitive to allosteric inhibition by high malate concentrations [169,170]. While class 1 PEPCs are constitutively expressed in the cytosol, the BPEPC is associated with the outer mitochondrial surface. This location is in line with a central role of this enzyme in collaboration with CA in the efficient fixation of respiratory CO_2_ and the anaplerotic supply of organic acids to the Krebs cycle [171]. This is especially relevant in developing seeds that store fatty acids such as castor bean seeds [172]. PEPC activity also plays a central role in symbiotic N_2_ fixation in root nodules (see Figure 2) where it provides OAA for nitrogen assimilation and malate for the bacteroids [173].

### 3.2. Carbon Concentration Mechanisms (CCM) in Terrestrial Plants

Under certain environmental conditions, CO_2_ may become a limiting factor for photosynthesis not only in cyanobacteria, algae, and aquatic macrophytes where CCMs have been intensively studied but also in terrestrial higher plants. High-temperature favoring photorespiration and drought imposes an increase of stomatal resistance. These are the main factors limiting CO_2_ availability for RuBisCo in land plants [174].

Long distance transport of HCO_3_^−^ from roots to leaves usually makes only a small contribution to C_inorg_ for photosynthesis (see Section 2.6). Exceptions are aquatic plants in the Lycophyta genus Isoetes and the non-stomatous land plant Stylites. They acquire all C_inorg_ for photosynthesis from the soil through the roots and recycle carbon by CAM [175]. Other terrestrial plants take up most of the C_inorg_ in the form of CO_2_ through stomata of the leaves. This CO_2_ diffuses into the chloroplast where it is assimilated by RuBisCO, which forms phopshoglycerate (PGA) as the first stable product of C_inorg_ assimilation. After activation with ATP and reduction by NADPH provided by the light–driven chloroplastic electron transport, PGA forms phosphoglyceraldehyde, which is the first sugar molecule of the photosynthetic carbon metabolism. Most terrestrial plants fix CO_2_ directly onto ribuslose-bis-phosphate. In contrast to plants with this so-called C_3_-type photosynthesis, plants with C_4_-type photosynthesis convert CO_2_ entering through the stomata into the mesophyll cells to HCO_3_^−^ using a cytosolic βCA. This C_inorg_ in the form of HCO_3_^−^ is initially fixed by PEP carboxylase in the cytosol of the outer mesophyll cells of the leaves. In this case, OAA is a first stable organic compound. Oxalacetate is either reduced to malate or transformed by transamination to aspartate. Malate or aspartate are the molecules that transfer the newly fixed carbon to the inner bundle sheet cells of the leaves where decarboxylation provides CO_2_, which is the substrate for Rubisco [176]. While CA activity is high in C_3_ chloroplasts where it facilitates the availability of CO_2_ for RuBisCo, the absence of CA activity from bundle sheet cells seems essential for the C_4_ mechanism [177].

This CCM around RuBisCo in C_4_ plants is considered an evolutionary adaptation to reduce the oxygenase activity of RuBisCo, which inhibits photorespiration and is especially enhanced under high temperature in tropical or subtropical areas [178]. However, C_4_-type photosynthesis can also be induced in certain amphibious plant species such as *Eleocharius vivipara* [179] under conditions of leaf emergence under dry conditions. Extreme adaptation to drought is observed in many CAM plants, which capture CO_2_ during the night when a lower temperature and a higher relative humidity in the atmosphere reduces transpiratory water loss. During the dark period, this C_inorg_ is fixed in the form of HCO_3_^−^ by PEP carboxylase and stored in large vacuoles mostly in the form of malate. The CO_2_ for fixation with RuBisCo is obtained by decarboxylation of malate during the following day-light period [180].

Limitations of CCMs in higher plants, especially of the C_3_- type of photosynthesis, and advances in our understanding of CCMs in cyanobacteria and microalgae like *C. rheinhardii* have promoted genetic engineering approaches to introduce efficient CCM into crop plants for increasing yield. Different approaches include manipulation of photorespiration, C_3_ to C_4_ engineering, and introduction of CCMs of cyanobacteria of *C. rheinharddii* into C_3_ crops. This well-known topic has recently been reviewed in detail by Mackinder [181] who identified gaps in our knowledge on bicarbonate transporter structure, functioning, and localization as important constraints that need priority attention for successful development of CCM engineered plants.

### 3.3. CO_2_/Bicarbonate Signalling in Stomatal Guard Cells

**Figure 3:**
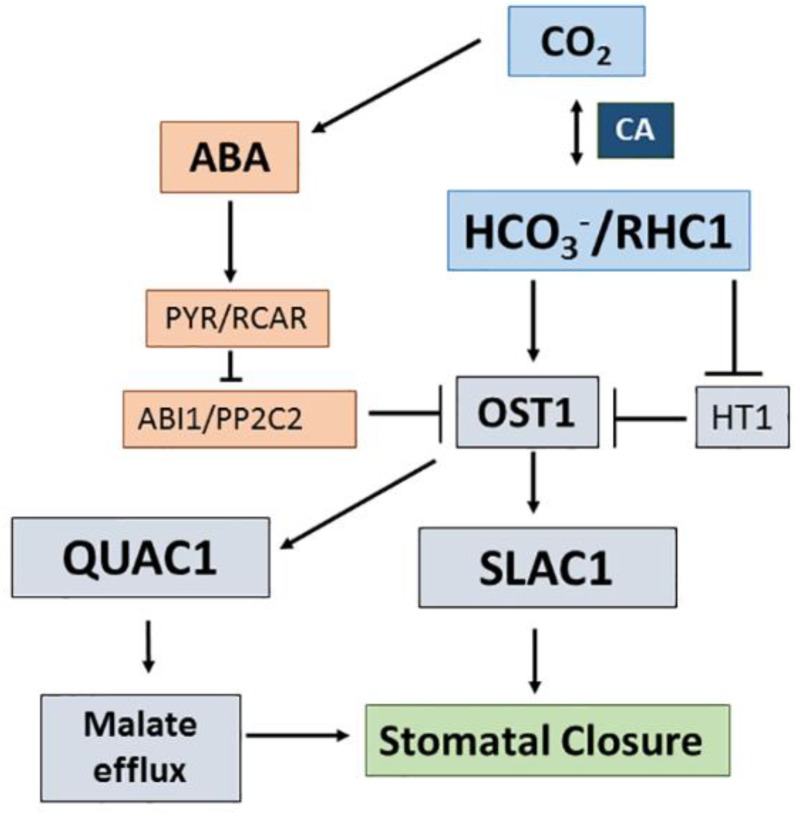
Mechanisms of stomatal closure induced by high CO_2_ or HCO_3_^−^ concentrations according to [183,184]. CA, carbonic anhydrase; ABA, abscisic acid; ABA receptor, PYR/RCAR, Pyrabactin Resistance (PYR) Regulator Component of ABA Receptor (RCAR); ABI1/PP2C2, Abscisic acid Insensitive Protein Phosphates C2; RHC1 Resistant to High CO2, MATE-type transporter specific activated by HCO_3_^−^; HT1, High Leaf Temperature kinase; OST1, Open Stomata1 protein kinase; SLAC1, Slow Anion Channel1; QUAC1, Quick Anion Channel1.

Regardless the type of photosynthesis, C_3_, C_4_, or CAM, the CO_2_ flux from the atmosphere into the plants is regulated by the stomatal opening and closure due to turgor changes in the stomatal guard cells. These changes are strictly controlled by multiple external and internal factors. Among those, the binomial CO_2_/HCO_3_^−^ plays a central role (see Figure 3). High CO_2_ promotes stomatal closure, which is brought about by the activation of efflux anion channels: SLAC1 (S-type) facilitating Cl^−^ or NO_3_^−^ efflux and R-type (AtALMT12/QUAC1 in *A. thaliana*) for malate efflux (see Section 2.6). The signal for stomatal closing in response to high CO_2_ seems to be a combination of alkaline pH, high Ca^2+^, and high HCO_3_^−^ in the cytosol [182]. The carbonic anhydrase double mutant *ca1ca4* does not show any effect on stomatal conductance when CO_2_ concentration is changed from 100 ppm to 80 ppm [183]. This points to HCO_3_^−^ being the key signal. Abscisic acid (ABA) dependent and ABA-independent mechanisms seem to operate in stomatal closure under a high amount of CO_2_ (see Figure 3). OST1 (Open Stomata 1) is a positive regulator of the anion efflux channels. In the ABA-independent signaling pathway, a high amount of HCO_3_^−^ activates a MATE–like transporter protein (RHC1, Resistance to High CO_2_), which acts as a positive regulator of OST 1 by inhibiting HT1 (High Leaf Temperature) known as a protein kinase that inactivates OST1 [183,184].

## 4. Plant Response to Bicarbonate-Rich Soils

It is common knowledge that limestone soils containing high carbonate/bicarbonate concentrations restrict the performance of calcifuge plant species and limit yield especially in iron-inefficient crops such as certain varieties of citrus, peach, pear, or soybeans suffering from lime-induced chlorosis [185,186]. Low pH leads to low availability of essential nutrients (especially Fe, Zn, and P) and high Ca soil concentrations are considered the main constraining factors. However, HCO_3_^−^ at concentrations occurring in the solution of limestone soils can inhibit root growth in sensitive plant species like the calcifuge grass *Deschampsia caespitosa* [187]. However, dicots like peas, beans, or sunflowers suffer more intense root growth inhibition due to CO_2_ and/or HCO_3_^−^ than the monocots barley and oats [123]. Recently soil carbonate has been identified as a main selection factor that drives local adaptation in natural populations of *A. thaliana*, which is a calcifuge species able to colonize soils with moderate carbonate contents [188]. 

Currently, no HCO_3_^−^ transporter has been characterized in higher plants (see Section 2.6). Nonetheless, HCO_3_^−^-induced root growth inhibition is paralleled by enhanced root production of organic acids especially malate, succinate, and citrate [189]. This suggests that excess HCO_3_^−^ enters the root and is metabolized by CA and PEP, which yields enhanced organic acid levels. However, CO_2_ diffusing from the soil’s atmosphere into the root can also be transformed inside the root into HCO_3_^−^ by CA. Bicarbonate can be released again into the soil rhizosphere, which contributes to the plant’s cation-anion balance. Especially under conditions of high nitrate uptake, enhanced HCO_3_^−^ efflux from the roots has been claimed to contribute to the characteristic alkalinization of the rhizosphere when nitrate is the main N source for the plants [190,191]. In fact, in maize and a tomato, the sum of K^+^ and NO_3_^−^ uptake and the HCO_3_^−^ efflux have been reported to be in electrical equilibrium [192]. However, no selective bicarbonate efflux transporters in plant roots have been reported and alkalinization can also be a consequence of either or both OH^−^ release or H^+^ uptake in cotransport with nitrate [193]. Actually, root supply of low HCO_3_^−^ concentrations tended to increase rather than decrease root nitrate uptake in *Populus canescens*. Exposure to 1 mM external NaHCO_3_ enhanced both nitrate reduction and assimilation as well as exported nitrogen to the shoots of poplar plants [194]. Higher HCO_3_^−^ concentrations cause net K^+^ and NO_3_^−^ efflux as well as accumulation of organic acids, mainly malate, in the roots [195]. To what extent dark fixation of C_inorg_ entering the roots plays a role in the carbon budget of terrestrial plants has been considered mainly in relation to lime-induced chlorosis in calcifuge plants. This type of chlorosis affects sensitive plant species when growing on carbonate-rich soil and may reflect an interference of HCO_3_^−^ in the mechanisms of Fe acquisition and transport [196]. Key processes potentially impaired by HCO_3_^−^ include the dicots’ strategy 1 such as the acidification of the rhizosphere due to the strong buffer ability of HCO_3_^−^ and the reduction of FeIII to FeII by ferric reductase, which operates optimally at acid pH [197]. The induction of root exudation of phenolic substances is not affected and is even stimulated by HCO_3_^−^. Induction of root accumulation and exudation of coumarin-type phenolics with high affinity for Fe has been reported as a response to Fe-deficiency under high pH conditions in *A. thaliana* [198]. An *A. thaliana* population which is naturally adapted to moderate soil carbonate had higher rates of coumarin root exudation than a sensitive population [188]. Furthermore, prevention of the imbalance of organic acid concentrations caused by dark fixation of HCO_3_^−^ and shifting of C_org_ into the shikimate pathway for the production of phenolic compounds has been reported as a mechanism of the extreme HCO_3_^−^ tolerance in *Parietaria difusa* [199]. In the view of the multiple implications of HCO_3_^−^ in plants’ metabolism, breeding programs for better crop yield on carbonate-rich soil would greatly benefit from the characterization at the genetic and molecular level of the of bicarbonate uptake and efflux mechanisms in higher land plants. 

## 5. Conclusions

During the last decade, there has been significant progress of our knowledge on the mechanisms of HCO_3_^−^ transport and CCM in cyanobacteria, algae, and seagrass species due to improved genetic and molecular tools and electrophysiological approaches. In contrast, in higher land plants, no HCO_3_^−^ transporter has been characterized so far. Advanced knowledge of the metabolic use of HCO_3_^−^ in terrestrial plants has mainly been made in relation to C_4_ and CAM metabolism including the genetic and molecular characterization of CAs and PEPC involved. However, there are still important gaps in our knowledge about the mechanisms of compartmentation and regulation especially regarding the complex interactions between light and dark fixation of C_inorg_, the recycling of respiratory and photorespiratory CO_2_, and the importance of anaplerotic supply of organic acids to the Krebs cycle. Filling these gaps is essential for progress in both genetic engineering approaches for transferring CCMs from cyanobacteria or microalgae to higher plants and breeding for bicarbonate tolerance in crops sensitive to lime-induced chlorosis.

## Figures and Tables

**Table 1:** C_inorg_ uptake mechanisms proposed for several seagrass species based on their photosynthetic sensitivity to TRIS and AZ. Question mark (?) denotes that the mechanism is partially supported by available evidences.

C_inorg_ Uptake Mechanism	AZ	TRIS	Seagrass Species	References
**Apoplastic dehydration of HCO_3_^−^ catalysed by CA**	+	−	*Zostera marina*	[93]
			*Cymodocea nodosa*	[96]
			*Halophyla ovalis*	[98]
			*Cymodocea serrulata*	[98]
			*Cymodocea rotundata*	[98]
			*Synringodium isoetifolium*	[98]
			*Halodule wrightii*	[98]
			*Thalassia hemprichii*	[98]
			*Thalassodendron ciliatum*	[98]
			*Enhalus acoroides*	[98]
			*Posidonia australis*	[102]
**Apoplastic dehydration of HCO_3_^−^ in acid regions**	+	+	*Halophila stipulacea*	[88]
			*Rupia maritima*	[88]
			*Cymodocea nodosa* (?)	[96]
			*Cymodocea rotundata*	[98]
			*Synringodium isoetifolium*	[98]
			*Halodule wrightii*	[98]
			*Thalassia hemprichii*	[98]
			*Thalassodendron ciliatum*	[98]
			*Enhalus acoroides*	[98]
**Plasma membrane HCO_3_^−^/H^+^ symport**	−	+	*Posidonia oceanica*	[27] ^1^
			*Zostera marina*	[94]
			*Halophyla stipulacea*	[88]
			*Rupia maritima*	[88]
			*Cymodocea nodosa* (?)	[96]
			*Halophila ovalis*	[98]

+ sensitive; − insensitive; ^1^ Direct evidences for a plasma membrane HCO_3_^−^/H^+^ symport mechanism.

## Abbreviations

ABA	Abscisic acid
CCM	Carbon concentration mechanism
CA	Carbonic anhydrase
PEPC	Phosphoenolpyruvate carboxylase
SLAC	Slow anion channel
SLC	Solute carriers
QAC	Quick anion channel
